# Association between sarcopenia and prognosis of hepatocellular carcinoma: A systematic review and meta-analysis

**DOI:** 10.3389/fnut.2022.978110

**Published:** 2022-12-14

**Authors:** Chuan Jiang, Yanyan Wang, Wei Fu, Guozhuan Zhang, Xiaoshan Feng, Xing Wang, Fang Wang, Le Zhang, Yang Deng

**Affiliations:** ^1^Department of Anoenterology, The Affiliated Hospital of Shandong University of Traditional Chinese Medicine, Shandong Provincial Hospital of Traditional Chinese Medicine, Jinan, China; ^2^Health Management Center, Qilu Hospital of Shandong University, Jinan, China; ^3^Department of Pain Management, Qilu Hospital of Shandong University, Jinan, China; ^4^Department of Endocrinology, Shandong Provincial Hospital Affiliated to Shandong First Medical University, Jinan, China; ^5^College of Clinical and Basic Medical Sciences, Shandong First Medical University and Shandong Academy of Medical Sciences, Jinan, China; ^6^School of Public Health, Shandong First Medical University and Shandong Academy of Medical Sciences, Jinan, China

**Keywords:** sarcopenia, skeletal muscle index, prognosis, hepatocellular carcinoma, meta-analysis

## Abstract

**Background:**

Sarcopenia, characterized by the loss of muscle mass, strength, and physical ability, occurs with aging and certain chronic illnesses such as chronic liver diseases and cancer. Sarcopenia is common in liver cirrhosis and hepatocellular carcinoma (HCC). Previous reports of association between sarcopenia and prognosis of HCC have been inconsistent. Therefore, the present systematic review and meta-analysis aimed to investigate the impact of sarcopenia on the survival of patients with HCC.

**Methods:**

A systematic literature search was conducted using PubMed, EMBASE, and Web of Science electronic databases from inception to May 1, 2022. We included retrospective or prospective studies investigating the association between sarcopenia and overall survival (OS) and/or progression free survival (PFS) of HCC. We applied the Quality in Prognosis Studies (QUIPS) instrument to evaluate the risk of bias and quality of included studies. The primary and secondary outcomes were the associations of sarcopenia with OS and PFS, respectively, expressed by a pooled hazard ratio (HR) and corresponding 95% confidence interval (CI). Subgroup analysis and sensitivity analysis were performed. We further evaluated the publication bias by the funnel plot and Begg’s test.

**Results:**

A total of 42 studies comprising 8,445 patients were included. The majority of included studies were at an overall low risk of bias. The pooled prevalence of sarcopenia was 39% (95% CI: 33–45%) (*n* = 8,203). Sarcopenia was associated with an increased risk of shorter OS, with a pooled adjusted HR of 1.84 (95% CI: 1.62–2.09). An independent association between sarcopenia and reduced PFS was observed (HR = 1.33, 95% CI: 1.12–1.56).

**Conclusion:**

The prevalence of sarcopenia was approximately 39% among patients with HCC. Sarcopenia was independently associated with reduced OS and PFS in HCC irrespective of treatment modalities. It is imperative that interventions aimed at alleviating sarcopenia and restoring muscle mass be implemented in order to improve the survival of patients with HCC.

**Systematic review registration:**

[https://www.crd.york.ac.uk/prospero/display_record.php?ID=CRD42022337797], identifier [CRD42022337797].

## Introduction

Liver cancer poses a major threat to the global cancer burden, and the number of deaths is estimated to be more than one million annually by 2030 (1, 2). Hepatocellular carcinoma (HCC) is the most common histologic type of liver cancer, accounting for approximately 90% of total cases (3). Curative therapies including hepatectomy, radiofrequency or microwave ablation, and liver transplantation are recommended as the first-line treatments when possible. Locoregional therapies such as transarterial chemoembolization (TACE), transarterial radioembolization (TARE), and radiation are associated with improved survival and quality of life for patients with unresectable HCC (4). However, curative therapies or locoregional therapies are not applicable to approximately 50% of HCC cases who are diagnosed at an advanced stage and have progression with transarterial therapies (5). For these patients with advanced HCC, sorafenib, lenvatinib, and atezolizumab combined with bevacizumab have been approved as the first-line systemic therapy, and regorafenib, cabozantinib and ramucirumab are second-line treatment options (6). The long-term prognosis of HCC patients is related to various factors, mainly represented by liver functional reserve, tumor size, treatment modalities, and Barcelona-Clínic Liver Cancer (BCLC) stage. Furthermore, maintenance of nutritional balance and physical ability is also an important factor in improving the prognosis of patients with advanced HCC (7).

Sarcopenia, characterized by low muscle mass in addition to impaired muscle strength and physical ability, is usually encountered in aging and patients with chronic illnesses such as chronic obstructive pulmonary disease, chronic renal failure, and cancer (8, 9). In recent years, the clinical significance of sarcopenia in cancer has attracted increasing attention. The associations between sarcopenia and the prognosis in patients with gastric cancer (10), colorectal cancer (11), lung cancer (12), ovarian cancer (13), and HCC (14, 15) have been investigated. For example, a cohort study revealed that sarcopenic patients with HCC undergoing TACE had a significantly poorer overall survival (OS) than those without sarcopenia (491 vs. 1,291 days, *P* = 0.017) (15). However, Ha et al. found that sarcopenia was not associated with OS in patients with newly diagnosed HCC (16). Thus, results of studies regarding the prognostic value of sarcopenia in patients with HCC remain inconsistent and even controversial. In this systematic review and meta-analysis, we aimed to determine the associations between sarcopenia and survival of patients with HCC following various treatment modalities, which may help identify sarcopenia as a prognostic factor for clinical decision making in patients with HCC.

## Materials and methods

This systematic review and meta-analysis was conducted in accordance with the Preferred Reporting Items for Systematic Reviews and Meta-Analyses (PRISMA) (17). The protocol was registered in the International Prospective Register of Systematic Reviews (PROSPERO) with the registration number CRD 42022337797.

### Search strategy

We systematically searched the PubMed, EMBASE, and Web of Science electronic databases for articles published from inception through May 1, 2022. The main search terms were described as follows: (“hepatocellular carcinoma” OR “HCC” OR “hepatoma”) AND (“sarcopenia” OR “sarcopenic” OR “skeletal muscle depletion”). Search results were restricted to articles published in English. The detailed search strategy is presented in Supplementary Table 1. Literature searching and screening were performed independently by two researchers (CJ and LZ), and disagreements between these two authors were resolved by a third researchers (YD).

### Selection criteria

We employed the populations, interventions, comparators, outcomes, and study designs (PICOS) outline to determine the eligibility of included publications as follows: (1) populations were patients diagnosed as HCC; (2) exposure was defined as sarcopenia; (3) compared to HCC patients without sarcopenia; (4) the outcomes were evaluated by prognostic indicators such as OS and/or progression free survival (PFS); (5) observational studies including retrospective and prospective studies were included. In addition, studies that met the following criteria were included for the qualitative and quantitative analysis: (1) patients diagnosed as HCC, (2) the measurement of sarcopenia or skeletal muscle mass was provided, (3) the association of sarcopenia with prognostic outcomes including OS and/or PFS were involved (4) hazard ratio (HR) with 95% confidence interval (CI) were provided or raw data were sufficient to calculate the HR and 95% CI, (5) retrospective or prospective study. The exclusion criteria included: (1) diagnostic criteria for sarcopenia were not provided, (2) sarcopenia was not regarded as a prognostic factor for OS and/or PFS in patients with HCC, (3) HR and corresponding 95% CI cannot be calculated from the data provided.

### Data extraction

Two researchers (CJ and YW) independently screened the titles and abstracts of articles which met the selection criteria. Full-text of the article was reviewed when its title or abstract was judged as eligible. Discrepancies between researchers were resolved by discussion with a third researcher (GZ). A predesigned electronic form was used to extract the following data from the included studies: last name of first author, publication year, study design, country of the study population, period of patient recruitment, baseline data of patients [i.e., number, sex, age, etiology, BCLC stage, TNM stage, and treatment], sarcopenia assessment and definitions (i.e., measurement methods, cut-point, and prevalence), and HR with corresponding 95% CI and adjustment factors in multivariate analysis of factors related to OS and/or PFS.

### Quality assessment

Two researchers (CJ and XF) independently employed the Quality in Prognosis Studies (QUIPS) Risk of Bias Assessment Instrument to assess the risk of bias for eligible studies (18, 19). The QUIPS instrument was used to evaluate the quality of prognosis studies, encompassing six domains: (1) study participation, (2) study attrition, (3) prognostic factor measurement, (4) outcome measurement, (5) adjustment for other prognostic factors, (6) statistical analysis and reporting. Each domain was rated as high, moderate, or low risk of bias. The judgment criterion of overall risk of bias was as follows: studies with ≤2 moderate-risk domains and ≥4 low-risk domains were considered as “overall low risk of bias,” those with >2 moderate-risk domains and <4 low-risk domains were classified as “overall moderate risk of bias,” while studies with ≥1 high-risk domain were defined as “overall high risk of bias” (20).

### Statistical analysis

The primary and secondary outcomes of this meta-analysis were the associations of sarcopenia with OS and PFS, respectively, expressed by a pooled HR and corresponding 95% CI. Cochran’s *Q* test and *I*^2^ statistics were performed to assess the heterogeneity across the included studies. A random effects model was chosen to estimate the pooled prevalence of sarcopenia and pooled HRs of associations between sarcopenia and OS or PFS. Sensitivity analysis by omitting one study at a time and then pooling the remaining studies was conducted to determine whether one study was a potentially important source of heterogeneity. We further evaluated the publication bias by the funnel plot and Begg’s test. If publication bias was observed, the trim-and-fill method was applied to estimate the potential influence of imputed unpublished studies with negative results on the outcome, and fail-safe number was calculated using the Rosenthal’s approach (21). All statistical analyses were performed using R software version 4.1.1 with “meta” and “metaphor” packages. A two-sided *P* value < 0.05 was regarded statistically significant.

## Results

### Literature search and study selection

**FIGURE 1:**
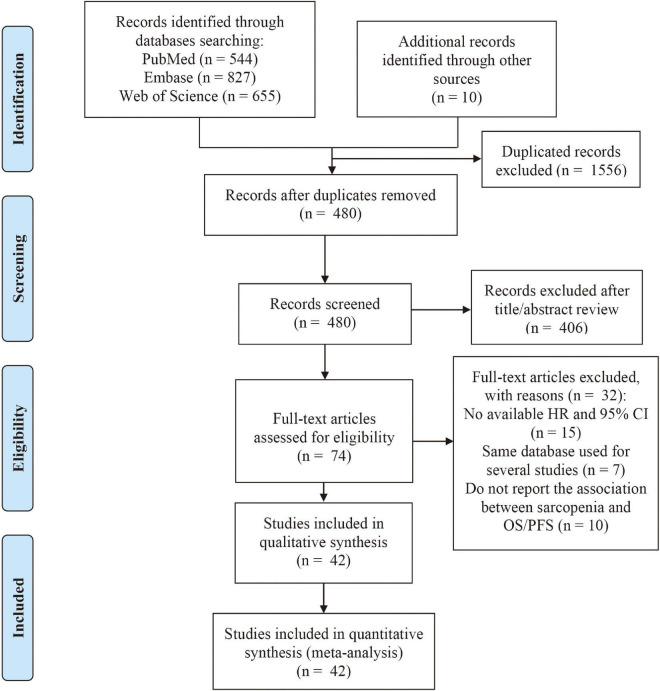
Flowchart of selecting and screening articles.

Figure 1 presents the flowchart of literature search and study selection according to the PRISMA guidelines. A total of 2,036 potentially relevant publications were identified in the literature search, of which 1,556 were excluded due to duplication. After screening the titles and abstracts of remaining 480 articles, 406 articles were removed for the following reasons: no clear definition of sarcopenia (*n* = 60), not human studies (*n* = 10), reviews/case reports/editorials (*n* = 336). After a full-text review of the 74 articles, another 32 articles were excluded for the following reasons: no available HR and 95% CI (*n* = 15), same database used for several studies (*n* = 7), do not report the association between sarcopenia and OS/PFS (*n* = 10). Finally, 42 articles were included in the qualitative and quantitative synthesis.

### Characteristics of included studies

Supplementary Table 2 outlines the main characteristics of the included studies. Overall, 42 studies comprising 8,445 patients (6,376 men and 2,069 women) were included. All included studies were published from 2013 onward. Regarding the research design, 38 studies were designed as retrospective studies, and four studies were conducted prospectively. Twenty-two studies were included from Japan (14, 22–42), six from Korea (16, 43–47), four from China (48–51), two from Egypt (52, 53), two from Germany (15, 54), two from Italy (55, 56), one each from Netherlands, Canada, United States of America (USA), and France (57–60). HCC patients were treated by hepatectomy, sorafenib, lenvatinib, TACE, yttrium-90 radioembolization, RFA, or the combination of these. Six methods for sarcopenia assessment were reported, including computed tomography (CT)-based skeletal muscle index (SMI), psoas muscle index (PMI) and total psoas volume (TPV) at the third lumbar vertebra (L3) level, CT based transverse psoas muscle thickness per body height (TPMT/BH) and intramuscular adipose tissue content (IMAC) at the level of umbilicus, and magnetic resonance imaging (MRI) derived fat-free muscle area (FFMA) at the level of the origin of the superior mesenteric artery. SMI is the most widely used index and sarcopenia is defined as SMI < 42 cm^2^/m^2^ for men and <38 cm^2^/m^2^ for women based on the guideline proposed by Japan Society of Hepatology (JSH) (61). Among twenty-seven studies using SMI, eight studies used the cut-points proposed by JSH (14, 22, 32, 35, 42, 45, 49, 52), six studies employed the Martin cut-points (41, 53, 55, 57, 58, 60), four studies applied the Vledder cut-points (27, 28, 34, 38), and their own cut-points were measured in other studies. HR was estimated by univariate and multivariate Cox proportional hazards regression to investigate the influence of sarcopenia on OS and/or PFS of HCC patients in the included studies.

### Quality assessment

Supplementary Table 3 presents the risk of bias of included studies using the QUIPS tool. Because most of the included studies were designed as retrospective studies, the risk of bias among these studies were regard as moderate in the study participation domain. Moreover, the moderate risk of bias was determined to be due to study attrition in eight studies, adjustment for other prognostic factors in eight studies, statistical analysis and reporting in five studies. In general, of the 42 included studies, 34 studies were at an overall low risk of bias (14–16, 22–25, 28–32, 34, 35, 37, 39–44, 46, 47, 49–59), while eight were at an overall moderate risk of bias (26, 27, 33, 36, 38, 45, 48, 60).

### Prevalence of sarcopenia

**FIGURE 2:**
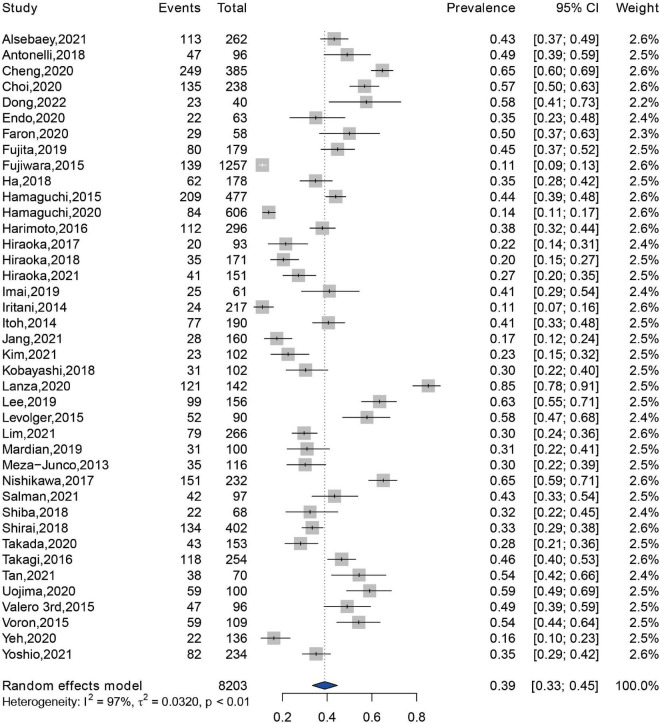
Forest plot showing the pooled prevalence of sarcopenia among patients with hepatocellular carcinoma.

Prevalence of sarcopenia were reported in 40 of the 42 eligible studies, with a sample size of 8,203 patients (14, 16, 22–60). The pooled prevalence of sarcopenia was 39% (95% CI: 33–45%) in the total patients (Figure 2). There was a highly significant heterogeneity in the prevalence of sarcopenia among these studies (*Q* = 1,389.98, *I*^2^ = 97.2%, *P* < 0.01). Subgroup analysis revealed that there were significant differences in the pooled prevalence among different methods for sarcopenia assessment (χ^2^ = 9.00, *P* = 0.01). The pooled prevalence of sarcopenia was 40% (95% CI: 33–47%) when assessed by SMI at L3 level, 31% (95% CI: 23–41%) by PMI at L3 level, and 52% (95% CI: 42–62%) by other methods, respectively (Supplementary Figure 1). When grouped by the location of individual studies, the pooled prevalence was 36% (95% CI: 30–42%) among studies conducted in Asia, 60% (95% CI: 45–74%) among studies conducted in Europe, 39% (95% CI: 22–58%) among studies conducted in North America, respectively (Supplementary Figure 2).

### Association between sarcopenia and OS

**FIGURE 3:**
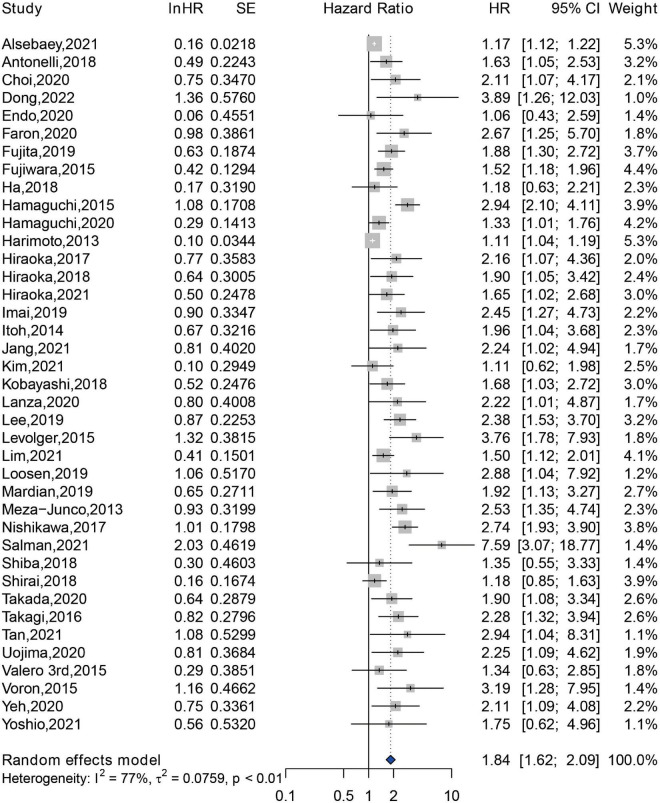
Forest plot of the pooled hazard ratio for association between sarcopenia and overall survival in patients with hepatocellular carcinoma.

A total of 39 included studies reported the association between sarcopenia and OS of HCC patients following various treatment modalities, with a sample size of 7,547 patients (14–16, 22–32, 34–46, 48–60). The result demonstrated that sarcopenia was associated with an increased risk of shorter OS, with a pooled adjusted HR of 1.84 (95% CI: 1.62–2.09) (Figure 3). A significant difference was observed in the test for heterogeneity, and a random effects model was conducted (*Q* = 166.81, *I*^2^ = 77%, *P* < 0.01). Subgroup analysis was performed according to the methods for sarcopenia assessment, and the pooled HRs of studies assessed by SMI at L3 level, by PMI at L3 level, and by other methods were 1.80 (95% CI: 1.53–2.11), 1.80 (95% CI: 1.48–2.18), and 2.39 (95% CI: 1.53–3.73), respectively (Supplementary Figure 3). In addition, we performed a subgroup analysis according to location of study. The result revealed that sarcopenia was an independent predictor of shorter OS among studies conducted in Asia (HR = 1.77, 95% CI: 1.54–2.03), in Europe (HR = 2.40, 95% CI: 1.72–3.34), and in North America (HR = 1.91, 95% CI: 1.03–3.54) (Supplementary Figure 4).

### Association between sarcopenia and PFS

**FIGURE 4:**
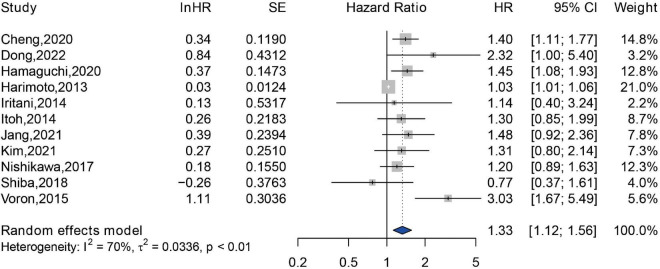
Forest plot of the pooled hazard ratio for association between sarcopenia and progression free survival in patients with hepatocellular carcinoma.

A total of 2,295 patients from 11 studies were included in the analyzing the impact of sarcopenia on the PFS (26–28, 33, 34, 37, 38, 44, 45, 48, 49, 60). An independent association between sarcopenia and reduced PFS was observed (HR = 1.33, 95% CI: 1.12–1.56), and a substantial statistical heterogeneity was exhibited (*Q* = 37.24, *I*^2^ = 70%, *P* < 0.01) (Figure 4). Among these 11 studies, 10 studies were assessed by SMI at L3 level, and only one study was assessed by TPMT/BH at umbilical level, therefore, subgroup analysis was not performed according to the methods for sarcopenia assessment. Additionally, 10 studies were conducted in Asia, and only one study was conducted in Europe. Thus, subgroup analysis of association between sarcopenia and PFS among different study locations was not available.

### Sensitivity analysis and publication bias

As shown in Supplementary Figures 5, 6, the results of sensitivity analysis demonstrated that no individual study had a significant influence on the pooled HRs of the associations of sarcopenia with OS and PFS, indicating that the pooled results were robust.

**FIGURE 5:**
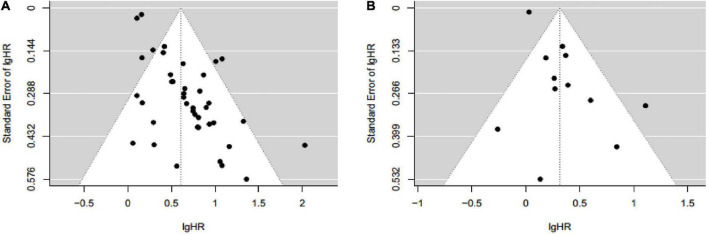
Funnel plot for assessing publication bias between sarcopenia with overall survival **(A)** and progression free survival **(B)** in patients with hepatocellular carcinoma.

The funnel plot for assessing publication bias between sarcopenia with OS was asymmetrical on visual evaluation, indicating a potential risk of publication bias, which was consistent with the result of Begg’s test (*P* = 0.044; Figure 5A). The trim-and-fill method was used to generate symmetrical funnel plot through incorporating 17 imputed unpublished studies with negative findings (Supplementary Figure 7). After trim-and-fill method, the pooled HR was 2.10 (95% CI: 1.90–2.30), which was similar to our result (HR = 1.84, 95% CI: 1.62–2.09). Furthermore, fail-safe number calculated using the Rosenthal’s approach was 3,476, suggesting that it was difficult to refute our results about the association between sarcopenia with OS. The funnel plot of association between sarcopenia and PFS was visually symmetrical (Figure 5B), and this result was confirmed by the Begg’s test (*P* = 0.091).

## Discussion

Sarcopenia was first proposed by Rosenberg to describe the loss of muscle mass with aging in 1988, and it was judged by an index calculated as appendicular skeletal muscle mass/the square of height (62). The definition of sarcopenia has evolved in the past decades, and three most common diagnostic indicators include lean muscle mass, impaired muscle strength, and low physical performance (63). Sarcopenia is common in the natural aging, functional, metabolic, and immune disorders, muscle hypercatabolism during cancer, and toxicity due to anti-cancer therapy (9, 64). Previous studies have shown that sarcopenia may co-occur with cachexia, and these two syndromes overlap considerably, especially in aging patients (65, 66). Most cachectic patients are also sarcopenic, but most individuals with sarcopenia are not considered as being cachectic. It is indicated that sarcopenia can be considered as a component of cachexia (67). Traditionally, sarcopenia is regarded as an inevitable consequence of aging. Two common types of sarcopenia are primary sarcopenia associated with aging and secondary sarcopenia caused by acute and chronic disorders which are related to muscle wasting, including chronic liver diseases (68). Chronic liver diseases are characterized by a progression from hepatitis, cirrhosis to hepatic decompensation or HCC (69). The prevalence of sarcopenia among patients with cirrhosis and those with alcohol-related liver disease or Child-Pugh class C cirrhosis were 37.5 and 50%, respectively (70). Patients with HCC are predisposed to the sarcopenia with prevalence reported between 11 and 85% in the studies included in this systematic review and meta-analysis. The pooled prevalence of sarcopenia among patients with HCC was 39% (95% CI: 33–45%) in this study. In addition, the varying prevalence was possibly attributed to the different assessment methods for sarcopenia and heterogeneous populations.

This assessment methods for sarcopenia were diverse and not yet standardized. The most common assessment method used in the included studies was cross-sectional CT-based SMI at the L3 level, and cut-points ranged from 36 to 55 cm^2^/m^2^ in men and 29 to 39 cm^2^/m^2^ in women (41, 42, 60). Cross-sectional imaging with CT or MRI is the conventional technique for diagnosis, staging, surveillance, and treatment response of HCC (71). Hence, it is available and reasonable to simultaneously evaluate muscle condition and prognosis in patients with HCC. The methods to define sarcopenia in several studies enrolled in this meta-analysis did not measure muscle strength or physical function. This is because sarcopenia was commonly used in cancer to denote low muscle mass without a measure of strength, and most of the included studies employed a retrospective design and did not include measurements of muscle strength or physical function at the data collection stage (7). Furthermore, there are several variations in the diagnosis of sarcopenia due to the different diagnostic criteria used, differences in the measurement methods used to assess muscle mass, differences in the cut-points applied, and heterogeneous study populations in the included studies. These could all contribute to the heterogeneity identified among studies (72–74). Further studies regarding the optimal method and cut-point for diagnosing sarcopenia are needed.

Accumulating evidence suggests that sarcopenia has an unfavorable impact on the prognosis of patients with HCC (56–58). Regarding the prognosis of patients with cancer, the criteria for the effectiveness of cancer drug trials proposed by U.S. Food and Drug Administration include the prolonged survival and improved clinical symptoms after therapy (75). OS and PFS are good criteria for evaluating clinical outcomes of patients with cancer. Currently, a growing number of meta-analyses have concentrated on the association between sarcopenia and prognosis of patients with cancer. In ovarian cancer, sarcopenia defined by a low SMI was associated with reduced OS (HR = 1.11, 95% CI: 1.03–1.20) (76). Yang Deng et al. observed that sarcopenia predicted a shorter OS in patients with non-small cell lung cancer (NSCLC) (HR = 2.57, 95% CI: 1.79–3.68) and small cell lung cancer (HR = 1.59, 95% CI: 1.17–2.14), but sarcopenia was not associated with PFS in NSCLC patients (HR = 1.28, 95% CI: 0.44–3.69) (20). A meta-analysis of 6 studies comprising 5,497 patients with female breast cancer confirmed that sarcopenia was an independent predictor of higher risk of mortality (HR = 1.71, 95% CI: 1.25–2.33) (77). Among patients with colorectal cancer (CRC), sarcopenia was associated with postoperative complications, postoperative mortality, and prolonged length of stay. Moreover, CRC patients with sarcopenia had worse OS, disease-free survival, and cancer-specific survival, compared to those without sarcopenia (78). Here, we conducted this meta-analysis to confirm that sarcopenia predicted poor OS (HR = 1.84, 95% CI: 1.62–2.09) and poor PFS (HR = 1.33, 95% CI: 1.12–1.56) in HCC patients receiving diverse treatments.

The mechanisms through which sarcopenia is associated with poor survival of patients with HCC are not fully understood, but several potential mechanisms can be proposed. Firstly, skeletal muscle homeostasis is maintained by muscle hypertrophy, atrophy, and regeneration. The disequilibrium of homeostasis especially between hypertrophy and regeneration can result in sarcopenia. The main characteristics of sarcopenia are a loss in muscle mass, muscle strength, and functional ability (71). The skeletal muscle is responsible for glucose disposal, and a loss of muscle mass causes insulin resistance, which increases the production and biological activity of insulin-like growth factor 1 (IGF-1). IGF-1 regulates proliferation of hepatocytes via protein kinase B/mammalian target of rapamycin (AKT/mTOR) signaling pathway, which is associated with advanced pathological stage, high risk of recurrence, and poor prognosis of HCC (79). Secondly, cancer patients with sarcopenia characterized by impaired muscle strength and/or physical performance exhibit a poor response to cancer treatments, and are associated with an increased risk of disease progression (27, 80). Thirdly, myokines including myostatin, interleukin 6 (IL-6), follistatin are synthesized and secreted by muscle fibers, exert immunological and anti-inflammatory effects, and facilitate proinflammatory states of liver fibrosis, cirrhosis, and hepatocarcinogenesis (43). High levels of IL-6 and follistatin are regarded as poor prognostic factors for OS in patients with HCC (81).

The present study has both strengths and limitations. One strength was that we performed appropriate and comprehensive statistical analysis including sensitivity analysis and subgroup analysis to confirm the reliability and applicability of the results. In addition, the volume of data assessed within this meta-analysis is sufficient, with 8,445 participants involved in the 42 studies included. However, several limitations should be acknowledged. Firstly, studies included in this meta-analysis used different methods and cut-points to assess sarcopenia, resulting in significant heterogeneity in the pooled prevalence of sarcopenia and the association of sarcopenia with OS and PFS. Thus, we chose a random effects model for these analyses, and performed a subgroup analysis. Secondly, the majority of the included studies were retrospective (90.5%), and this meta-analysis might be susceptible to information bias and confounding bias. Thirdly, asymmetric funnel plot for OS indicates a potential risk of publication bias. To take this into account, we used the trim-and-fill method and calculated the fail-safe number to evaluate the impact of publication bias on the results about the association between sarcopenia with OS. Finally, this meta-analysis was restricted to articles published in English, and qualified articles in other languages were not included in the analysis, which might introduce bias.

## Conclusion

In summary, the prevalence of sarcopenia was approximately 39% among patients with HCC. Sarcopenia was considered as an unfavorable prognostic factor and was independently associated with reduced OS and PFS in HCC irrespective of treatment modalities. It is suggested that assessment and early detection of sarcopenia, and interventions including suitable physical exercise and supplemental nutrition should be implemented to improve the prognosis of patients with HCC. A consensus on the optimal method and cut-point to assess sarcopenia needs to be reached.

## Data availability statement

The original contributions presented in this study are included in the article/Supplementary material, further inquiries can be directed to the corresponding authors.

## Author contributions

CJ, LZ, and YD designed the study protocol and conducted the literature search. CJ, YW, WF, and GZ retrieved and selected the article. CJ, XF, XW, FW, and LZ conducted data extraction. CJ, YW, WF, GZ, and YD performed the statistical analysis of the data. CJ and YW wrote the manuscript draft. LZ and YD supervised the study. All authors contributed to the article and approved the submitted version.
