# Supplementary material for: Association between sarcopenia and prognosis of hepatocellular carcinoma: A systematic review and meta-analysis
Source: Front Nutr. 2022 Dec 14;9:978110. doi: 10.3389/fnut.2022.978110 (PMC9794869; doi:10.3389/fnut.2022.978110)
Supplement: Supplementary file 1 [file Data_Sheet_1.docx]

***Supplementary Materials***

**Association between** **Sarcopenia and Prognosis of Hepatocellular Carcinoma: A Systematic Review and Meta-Analysis**

Chuan Jiang^†^, Yanyan Wang^†^, Wei Fu^†^, Guozhuan Zhang^†^, Xiaoshan Feng, Xing Wang, Fang Wang, Le Zhang^*^ and Yang Deng^*^

**^*^Correspondence:**

Yang Deng: dengyang3417@126.com.

Le Zhang: sdzhangle@163.com

**
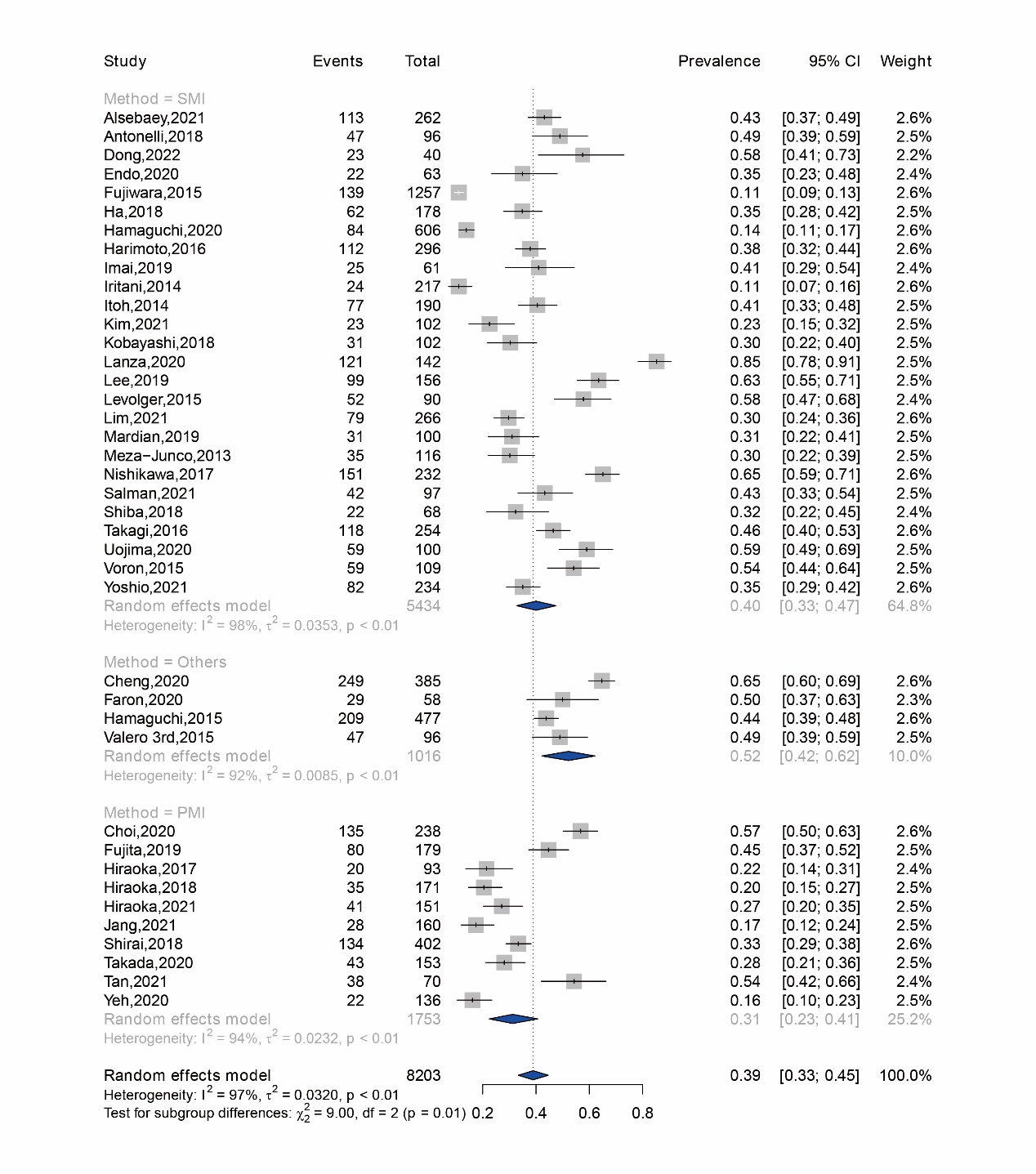
**

**Supplementary Figure 1.** Subgroup analysis for prevalence of sarcopenia in patients with hepatocellular carcinoma by different assessment methods


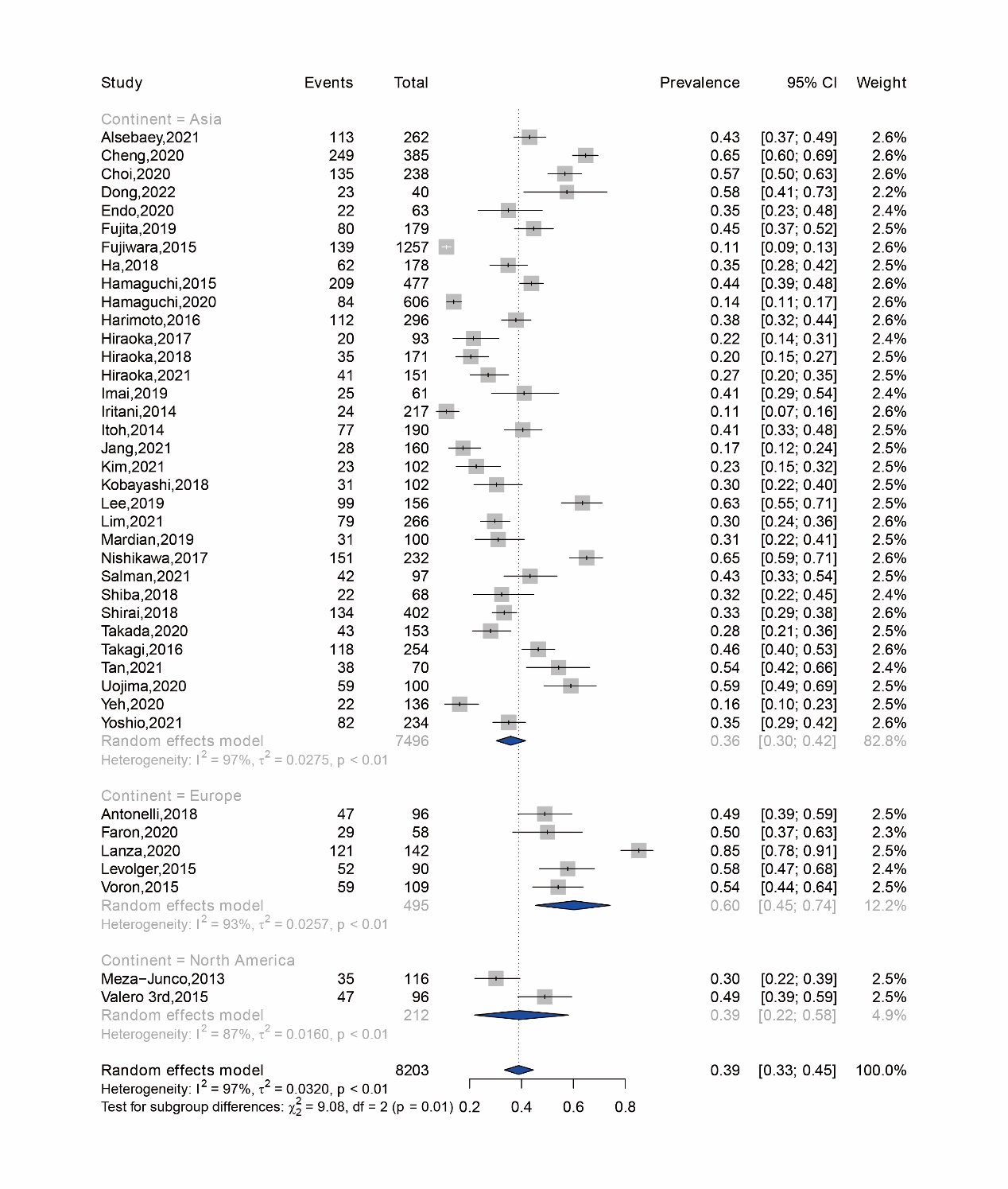


**Supplementary Figure 2.** Subgroup analysis for prevalence of sarcopenia in patients with hepatocellular carcinoma by the location of study


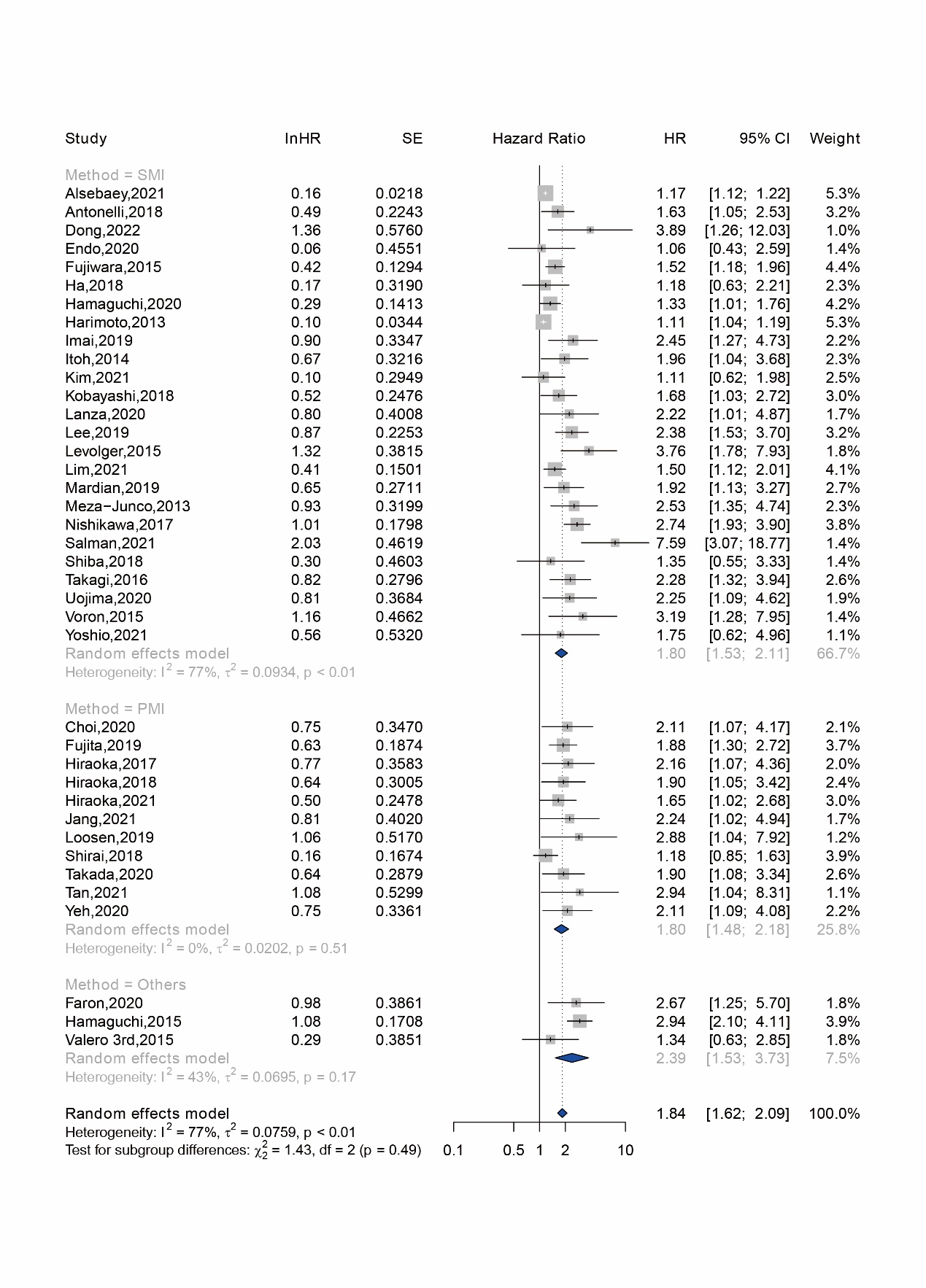


**Supplementary Figure 3.** Subgroup analysis of association between sarcopenia and overall survival in patients with hepatocellular carcinoma by different assessment methods


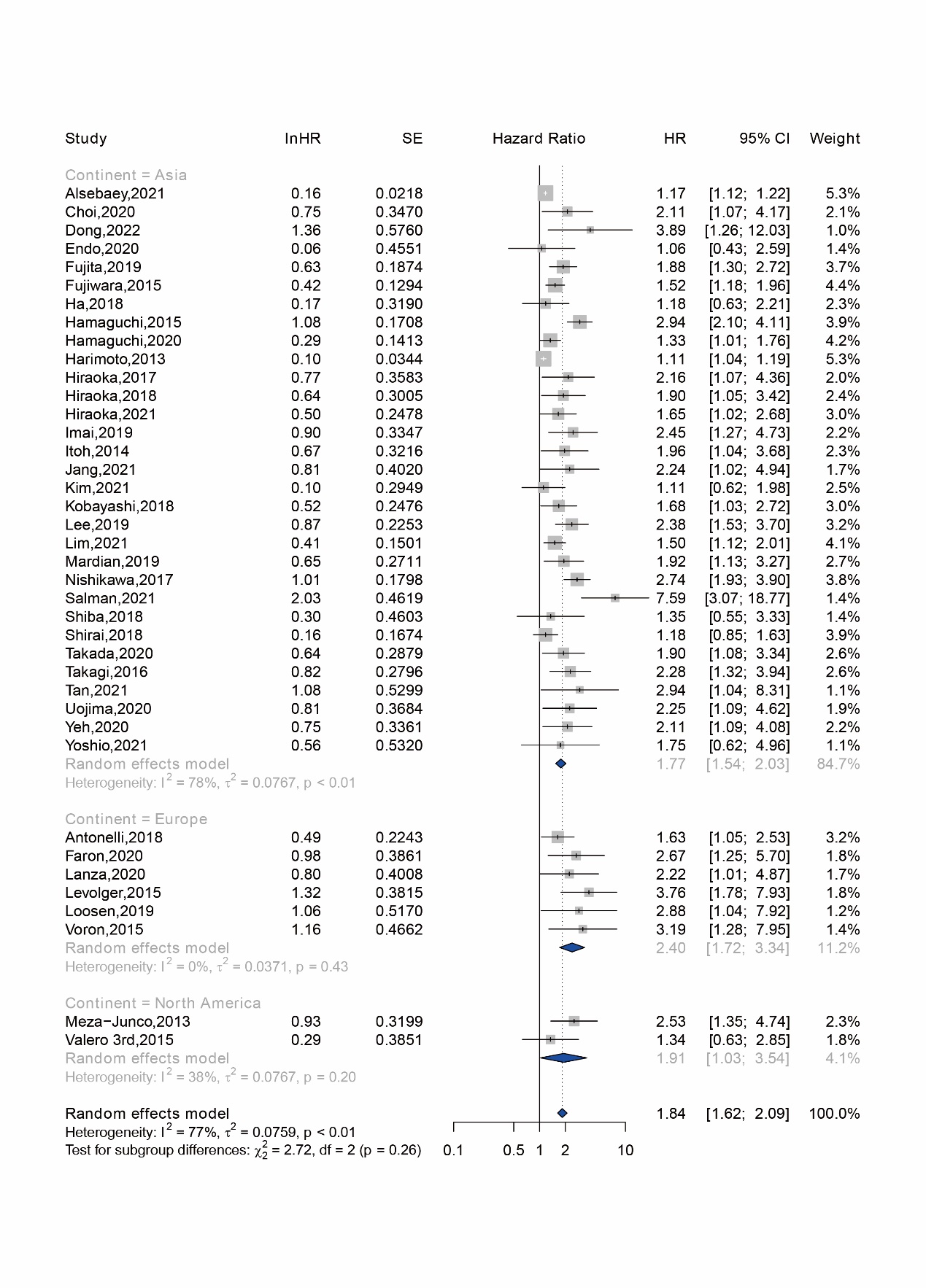


**Supplementary Figure 4.** Subgroup analysis of association between sarcopenia and overall survival in patients with hepatocellular carcinoma by the location of study


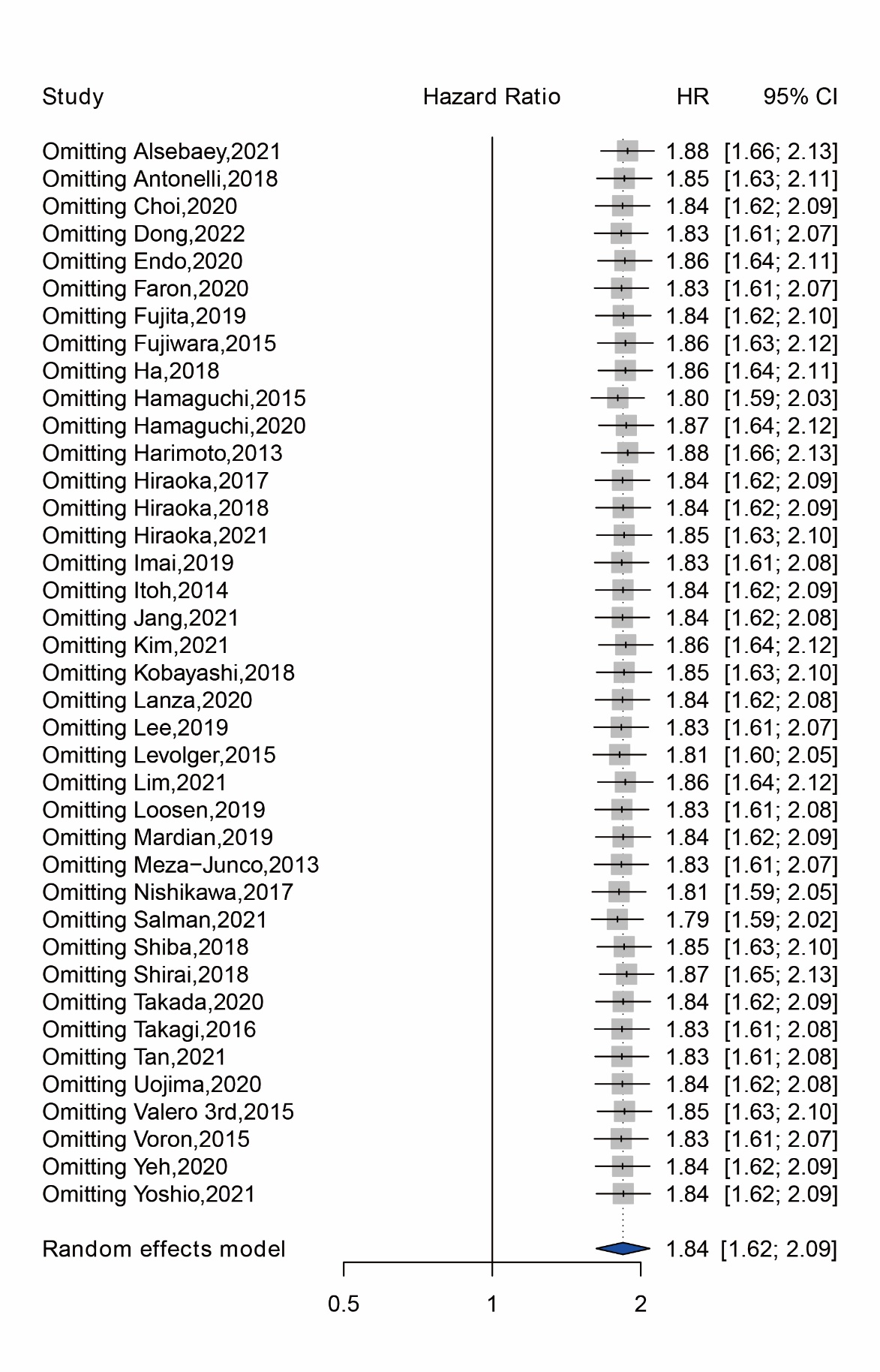


**Supplementary Figure 5.** Sensitivity analysis of association between sarcopenia and overall survival in patients with hepatocellular carcinoma based on leave-one-out method


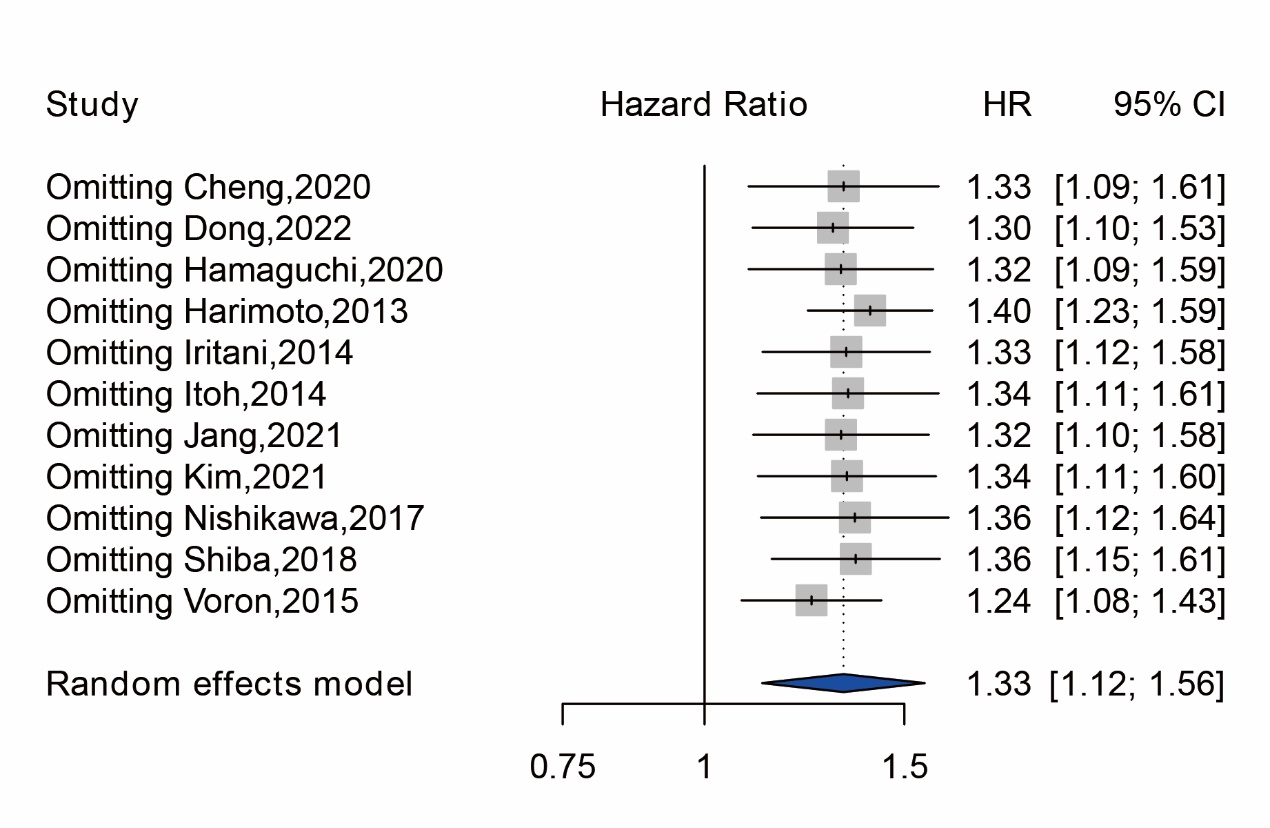


**Supplementary Figure 6.** Sensitivity analysis of association between sarcopenia and progression free survival in patients with hepatocellular carcinoma based on leave-one-out method


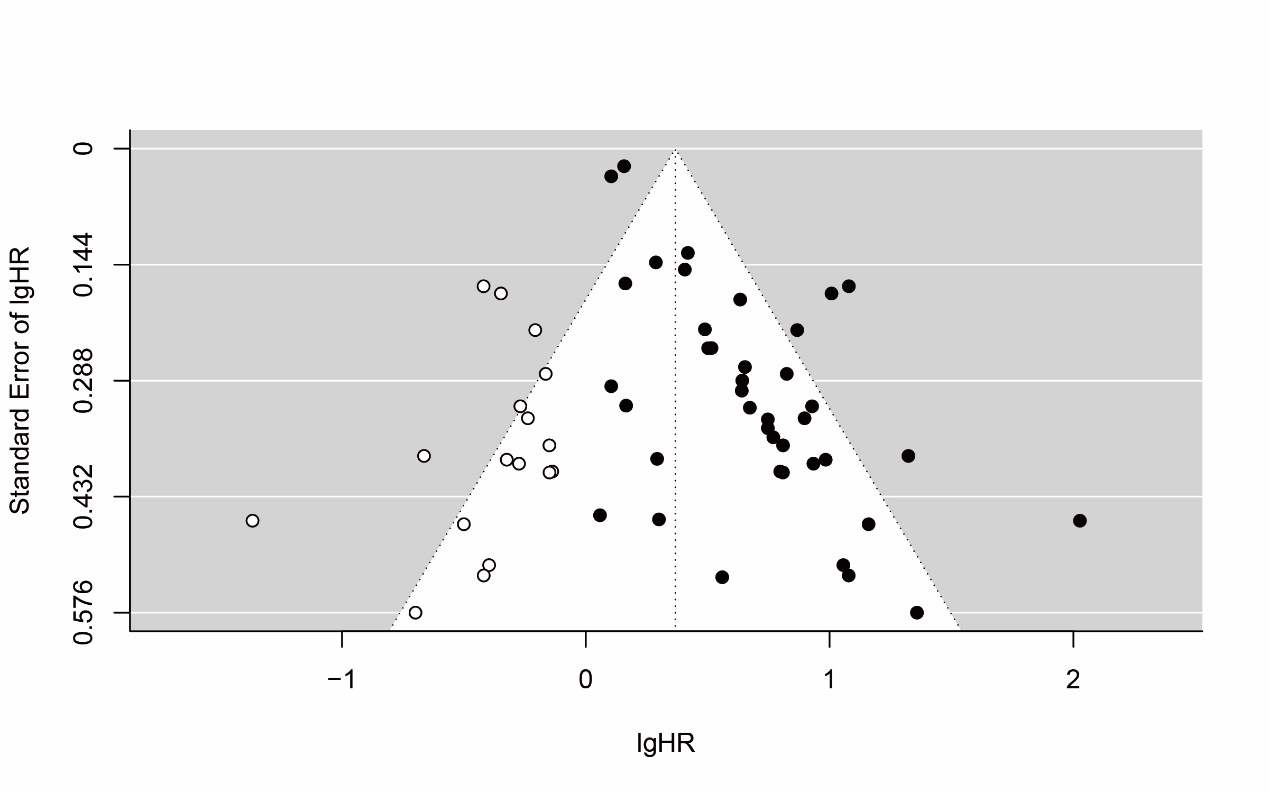


**Supplementary Figure 7.** Funnel plots with ‘trim-and-fill’ analyses for the meta-analysis between sarcopenia with overall survival in patients with hepatocellular carcinoma

**Supplementary Table 1.** Literature search strategies

| **Step** | **Search strategy** | **Number of literatures** |
| --- | --- | --- |
| ***PubMed*** | | |
| #1 | "hepatocellular carcinoma"[MeSH Terms] | 96,180 |
| #2 | "sarcopenia"[MeSH Terms] | 7,149 |
| #3 | hepatocellular carcinoma OR HCC OR hepatoma [Title/Abstract] | 153,526 |
| #4 | sarcopenia OR sarcopenic OR skeletal muscle depletion [Title/Abstract] | 255,531 |
| #5 | #1 AND #2 | 71 |
| #6 | #3 AND #4 | 554 |
| #7 | #5 OR #6 | 554 |
| #8 | #7 AND English[Language] | 544 |
| ***Embase*** | | |
| #1 | 'hepatocellular carcinoma'/exp | 186,382 |
| #2 | 'sarcopenia'/exp | 15,528 |
| #3 | 'hepatocellular and carcinoma':ab,ti OR 'HCC':ab,ti OR 'hepatoma':ab,ti | 187,592 |
| #4 | 'sarcopenia':ab,ti OR 'sarcopenic':ab,ti OR ' skeletal muscle depletion':ab,ti | 174,608 |
| #5 | #1 AND #2 | 354 |
| #6 | #3 AND #4 | 717 |
| #7 | #5 OR #6 | 841 |
| #8 | #7 AND [english]/lim | 827 |
| ***Web of Science*** | | |
| #1 | TS=(hepatocellular carcinoma OR HCC OR hepatoma OR (hepatocellular and carcinoma)) | 132,105 |
| #2 | TS=(sarcopenia OR sarcopenic OR skeletal muscle depletion) | 138,120 |
| #3 | LA=(English) | 29,557,947 |
| #4 | (#1 AND #2 AND #3) | 655 |

**Supplementary Table 2.** Main characteristics and results of the included studies

| **Study** | **Design** | **Country** | **Period of recruitment** | **No. of patients (male/female)** | **Age (years)^*^** | **Etiology** | **BCLC stage (0/A/B/C/D)** | **TNM stage (I/II/III/IV)** | **Initial treatment** | **Sarcopenia measurement** | **Criterion of cut-points** | **Cut-points** | **Sarcopenia case (%)** | **Outcomes** | **HR (95%CI)^#^** | **Adjustment factors** |
| --- | --- | --- | --- | --- | --- | --- | --- | --- | --- | --- | --- | --- | --- | --- | --- | --- |
| Alsebaey et al. 2021 [52] | Retrospective | Egypt | 2017-2020 | 262(182/80) | 59.61 ± 8.09 | HBV 17, HCV 233, viral negative 6, NASH 5, other 1 | 0/74/142/46/0 | NR | HCC awaiting liver transplantation | CT based SMI at L3 | JSH | Male: 50 cm^2^/m^2^, female: 39 cm^2^/m^2^ | 113(43.13) | OS | 1.17 (1.12-1.22) | MELD score, ALBI, BCLC B+C stage |
| Antonelli et al. 2018 [55] | Retrospective | Italy | 2008-2016 | 96(75/21) | 69 (30-87) | HBV 13, HCV 46, alcohol 16, NASH 11, other 10 | 0/0/22/74/0 | NR | Advanced HCC treated with sorafenib | CT based SMI at L3 | Martin | Male: 53 cm^2^/m^2^ for BMI ≥ 25 kg/m^2^, 43 cm^2^/m^2^ for BMI < 25 kg/m^2^, female: 41 cm^2^/m^2^ | 47(48.96) | OS | 1.63 (1.05-2.53) | Vascular invasion, MELD score |
| Cheng et al. 2020 [48] | Retrospective | China | 2012-2017 | 385(302/83) | 63.08 ± 12.79 | HBV 254, HCV 98, alcohol 23, other 10 | NR | NR | Advanced HCC after sorafenib failure | CT based TPMT/BH at umbilicus level | Original | 16.8 mm/m | 249(64.68) | PFS | 1.404(1.112-1.773) | Tumor size, AFP, Child–Pugh class, progressive disease, new extrahepatic mets |
| Choi et al. 2020 [43] | Prospective | Korea | 2012-2015 | 238(193/45) | 59 (53-69) | HBV 177, HCV 22, viral negative 39 | 43/95/43/57/0 | 111/43/80/4 | Hepatectomy 8, RFA 38, TACE 187, systemic therapy 3 | CT based PMI at L3 | EWGSOP | Male: 4.98 cm^2^/m^2^ female: 1.17 cm^2^/m^2^ | 135(56.72) | OS | 2.11(1.07-4.17) | AFP, follistatin, MELD score, ECOG PS, TNM stage |
| Dong et al. 2022 [49] | Retrospective | China | 2018-2020 | 40(37/3) | 59 (47-63) | HBV 35, HCV 3, viral negative 2 | 0/0/12/28/0 | 0/5/15/20 | Unresectable HCC treated with lenvatinib | CT based SMI at L3 | JSH | Male: 42 cm^2^/m^2^, female: 38 cm^2^/m^2^ | 23(57.50) | OS | 3.89(1.26-12.05) | Albumin, maximum tumor diameter, portal vein thrombosis |
|  |  |  |  |  |  |  |  |  |  |  |  |  |  | PFS | 2.32(0.997-5.405) | NR |
| Endo et al. 2020 [22] | Retrospective | Japan | 2018-2020 | 63(53/10) | 71 (50-86) | HBV 10, HCV 23, alcohol 17, other 13 | 0/0/19/44/0 | NR | Unresectable HCC treated with lenvatinib | CT based SMI at L3 | JSH | Male: 42 cm^2^/m^2^, female: 38 cm^2^/m^2^ | 22(34.92) | OS | 1.06(0.43-2.56) | modified ALBI, decreased grip strength |
| Faron et al. 2020 [54] | Retrospective | Germany | 2007-2014 | 58(45/13) | 68 ± 12 | HBV 11, HCV 11, alcohol 9, other 27 | 0/1/22/35/0 |  | Unresectable HCC receiving yttrium-90 radioembolization | MRI derived FFMA at the origin of superior mesenteric artery level | Original | Male: 3582 mm^2^, female: 2301mm^2^ | 29(50.00) | OS | 2.675(1.255-5.702) | ECOG PS, estimated liver tumor  burden |
| Fujita et al. 2019 [23] | Retrospective | Japan | 2006-2017 | 179(130/49) | 72 (64-78) | HBV 24, HCV 85, alcohol 39, NASH 26, other 5 | NR | 14/70/71/24 | HCC undergoing TACE and/or TAI | CT based PMI at L3 | JSH | Male: 6.0 cm^2^/m^2^ female: 3.4 cm^2^/m^2^ | 80(44.69) | OS | 1.884(1.305-2.720) | Child–Pugh class, TNM stage, AFP, PIVKA-II, progressive disease |
| Fujiwara et al. 2015 [24] | Retrospective | Japan | 2004-2009 | 1257(828/429) | 68.8 ± 9.2 | HBV 142, HCV 895, viral negative 207, other 13 | 181/588/427/47/14 | NR | Percutaneous therapy 538, TACE 40, percutaneous therapy plus TACE 144, no treatment 535 | CT based SMI at L3 | Original | Male: 36.2 cm^2^/m^2^, female: 29.6 cm^2^/m^2^ | 139(11.06) | OS | 1.52(1.18-1.96) | intramuscular fat deposition, visceral adiposity, viral status, platelet count, BCLC stage, previous treatment, AFP |
| Ha et al. 2018 [16] | Retrospective | Korea | 2007-2012 | 178(141/37) | 59.8 ± 12.3 | HBV 110, HCV 15, alcohol 27, unknown 26 | 0+A44/20/100/14 | NR | Untreated HCC | CT based SMI at L3 | Original | Male: 45.8 cm^2^/m^2^, female: 43.0 cm^2^/m^2^ | 62(34.83) | OS | 1.18(0.63-2.20) | Visceral to subcutaneous fat area ratio, AFP, tumor size, infiltrative tumor, BCLC stage |
| Hamaguchi et al. 2015 [25] | Retrospective | Japan | 2005-2014 | 477(389/88) | 67 ± 10 | HBV or/and HCV 319, other 158 | NR | 82/181/142/72 | HCC undergoing hepatectomy | CT based IMAC at umbilicus level | Original | Male: -0.324, female: -0.138 | 209(43.82) | OS | 2.942(2.117-4.136) | AFP, TNM stage |
| Hamaguchi et al. 2020 [26] | Retrospective | Japan | 2005-2016 | 606(484/122) | 68 (61-75) | HBV or/and HCV 392, other 214 | NR | I + II 361, III + IV 245 | HCC undergoing hepatectomy | CT based SMI at L3 | Original | Male: 40.31 cm^2^/m^2^, female: 30.88 cm^2^/m^2^ | 84(13.86) | OS | 1.333(1.007-1.752) | IMAC, VSR, TNM stage, AFP, Albumin |
|  |  |  |  |  |  |  |  |  |  |  |  |  |  | PFS | 1.447(1.074-1.913) | IMAC, VSR, AFP, TNM stage |
| Harimoto et al. 2013 [27] | Retrospective | Japan | 2004-2009 | 186(145/41) | 66.4 ± 10.4 | NR | NR | 29/95/49/13 | HCC undergoing hepatectomy | CT based SMI at L3 | Vledder | Male: 43.75 cm^2^/m^2^, female: 41.10 cm^2^/m^2^ | 75(40.32) | OS | 1.11(1.04-1.19) | Poor differentiation, MVI postoperative complications |
|  |  |  |  |  |  |  |  |  |  |  |  |  |  | PFS | 1.03(1.00-1.05) | ICGR15, DCP, TNM stage III + IV, intrahepatic metastases |
| Harimoto et al. 2016 [28] | Retrospective | Japan | 2004-2013 | 296(221/75) | NR | HBV 28, HCV 116, other 152 | NR | 48/156/74/17 | HCC undergoing hepatectomy | CT based SMI at L3 | Vledder | Male: 43.75 cm^2^/m^2^, female: 41.10 cm^2^/m^2^ | 112(37.84) | OS | 2.544(1.206-5.586) | Child–Pugh B, multiple tumor number, poor differentiation |
|  |  |  |  |  |  |  |  |  |  |  |  |  |  | PFS | 1.821(1.037-3.194) | TNM Stage III + IV, blood transfusion |
| Hiraoka et al. 2017 [29] | Retrospective | Japan | 2009-2015 | 93(81/12) | 68.3 ± 9.4 | HCV 56, HBV 18, HBV+HCV 2, alcohol 4, other 13 | NR | 0/10/28/55 | HCC treated with sorafenib | CT based PMI at L3 | Original | Male: 4.24 cm^2^/m^2^ female: 2.50 cm^2^/m^2^ | 20(21.51) | OS | 2.158(1.069-4.355) | DCP |
| Hiraoka et al. 2018 [30] | Retrospective | Japan | 2007-2015 | 171(130/41) | 70.4 ± 9.2 | HCV 96, HBV 17, alcohol 8, other 50 | NR | 17/104/39/11 | HCC undergoing hepatectomy | CT based PMI at L3 | Original | Male: 4.24 cm^2^/m^2^ female: 2.50 cm^2^/m^2^ | 35(20.47) | OS | 1.896(1.052-3.416) | Child-Pugh classification B, beyond Milan criteria |
| Hiraoka et al. 2021 [31] | Retrospective | Japan | 2018-2020 | 151(116/35) | NR | HCV 71, HBV 24, alcohol 24, other 32 | 0/2/52/96/1 | 2/16/50/83 | unresectable HCC treated with lenvatinib | CT based PMI at L3 | Original | Male: 4.24 cm^2^/m^2^ female: 2.50 cm^2^/m^2^ | 41(27.15) | OS | 1.652(1.017-2.686) | AFP, BCLC C/D stage |
| Imai et al. 2019 [32] | Retrospective | Japan | 2009-2017 | 61(54/7) | 67.3 ± 11.5 | HBV 14, HCV 28, other 19 | NR | 0/0/20/41 | HCC treated with sorafenib | CT based SMI at L3 | JSH | Male: 42 cm^2^/m^2^, female: 38 cm^2^/m^2^ | 25(40.98) | OS | 2.453(1.273-4.728) | change in SMI, change in SFMI, therapeutic effect |
| Iritani et al. 2014 [33] | Retrospective | Japan | 2006-2012 | 217(146/71) | 72 (27-90) | HBV 28, HCV 134, HBV+HCV 3, other 52 | NR | 52/71/66/28 | Hepatectomy 45, RFA 97, TACE or TAI 61, radiation 14 | CT based SMI at L3 | Original | Male: 36 cm^2^/m^2^, female: 29 cm^2^/m^2^ | 24(11.06) | PFS | 1.1425(0.3461-2.7821) | NR |
| Itoh et al. 2014 [34] | Retrospective | Japan | 2004-2009 | 190(146/44) | NR | NR | NR | NR | HCC undergoing hepatectomy | CT based SMI at L3 | Vledder | Male: 43.75 cm^2^/m^2^, female: 41.10 cm^2^/m^2^ | 77(40.53) | OS | 1.96(1.06-3.74) | Multiple tumors, MVI, intraoperative blood transfusion |
|  |  |  |  |  |  |  |  |  |  |  |  |  |  | PFS | 1.30(0.85-2.00) | NR |
| Jang et al. 2021 [44] | Retrospective | Korea | 2003-2011 | 160(120/40) | 55.19 ± 11.49 | HBV 125, HCV 12, viral negative 23 | 11/107/31/11/0 | 75/62/23/0 | HCC undergoing hepatectomy | CT based PMI at L3 | Original | Male: 3.33 cm^2^/m^2^ female: 2.38 cm^2^/m^2^ | 28(17.50) | OS | 2.245(1.021-4.937) | Low platelet count, High MELD-Na score, TNM stage II + III, VSR |
|  |  |  |  |  |  |  |  |  |  |  |  |  |  | PFS | 1.477(0.924-2.362) | NR |
| Kim et al. 2021 [45] | Retrospective | Korea | 2017-2018 | 102(87/15) | 61.3 (54-69) | HBV 78, HCV 7, alcohol 8, unknown 9 | NR | NR | HCC treated with nivolumab | CT based SMI at L3 | JSH | Male: 42 cm^2^/m^2^, female: 38 cm^2^/m^2^ | 23(22.55) | OS | 1.11(0.62-1.97) | NR |
|  |  |  |  |  |  |  |  |  |  |  |  |  |  | PFS | 1.31(0.80-2.14) | NR |
| Kobayashi et al. 2018 [35] | Retrospective | Japan | 2005-2015 | 102(70/32) | 69 (34-89) | HBV 11, HCV 50, alcohol 26, NASH 7, other 8 | NR | 11/22/46/23 | HCC undergoing TACE and/or TAI | CT based SMI at L3 | JSH | Male: 42 cm^2^/m^2^, female: 38 cm^2^/m^2^ | 31(30.39) | OS | 1.675(1.031-2.721) | AFP, maximum tumor diameter |
| Lanza et al. 2020 [56] | Retrospective | Italy | 2011-2019 | 142(110/32) | 73 ± 9.5 | HBV 7, HCV 65, alcohol 33, NASH 21, unknown 16 | 7/31/81/21/1, unknown 1 | NR | HCC undergoing TAE | CT based SMI at L3 | Original | Male: 55 cm^2^/m^2^, female: 39 cm^2^/m^2^ | 121(85.21) | OS | 2.22(1.01-4.86) | number of nodules |
| Lee et al. 2019 [46] | Retrospective | Korea | 2009-2016 | 156(128/28) | 59 (23-87) | HBV 113, HCV 14, viral negative 29 | NR | 3/15/48/90 | HCC undergoing radiotherapy | CT based SMI at L3 | Original | Male: 49 cm^2^/m^2^, female: 41 cm^2^/m^2^ | 99(63.46) | OS | 2.38(1.53-3.70) | ALBI score, total dose of radiotherapy |
| Levolger et al. 2015 [57] | Retrospective | Netherlands | 2002-2013 | 90(63/27) | 62 (22-86) | HBV 15, HCV 22, other 53 | 15/30/36/9/0 | NR | Hepatectomy 61, RFA 29 | CT based SMI at L3 | Original | Male: 52 cm^2^/m^2^, female: 39.5 cm^2^/m^2^ | 52(57.78) | OS | 3.756(1.778-7.932) | age, tumor size |
| Lim et al. 2021 [47] | Retrospective | Korea | 2007-2015 | 266(187/79) | 69.9 ± 9.5 | HBV 155, HCV 60, other 51 | 29/136/101/0/0 | NR | HCC undergoing TACE | CT based SMI at L3 | Original | Male: 49.6 cm^2^/m^2^, female: 43.1 cm^2^/m^2^ | 79(29.70) | OS | 1.501(1.118-2.014) | age, MELD score, size of tumor, albumin, platelet, BCLC A+B stage, objective tumor response |
| Loosen et al. 2019 [15] | Prospective | Germany | 2013-2018 | 56(44/12) | 65 (30-89) | NR | 0/19/33/4/0 | NR | HCC undergoing TACE | CT based PMI at L3 | Original | 13.39 cm^2^/m^2^ | NR | OS | 2.876(1.044-7.922) | Albumin |
| Mardian et al. 2019 [36] | Retrospective | Japan | 2016-2018 | 100(74/26) | 55.03 ± 11.20 | HBV 58, HCV 8, other 34 | 0/7/25/59/9 | 20/5/32/43 | HCC undergoing TACE or sorafenib 65, end-stage life support 35 | CT based SMI at L3 | Original | Male: 36.2 cm^2^/m^2^, female: 29.6 cm^2^/m^2^ | 31(31.00) | OS | 1.921(1.129-3.268) | Male, intramuscular fat deposition, BCLC B+C stage |
| Meza-Junco et al. 2013 [58] | Prospective | Canada | NR | 116(98/18) | 58.0 ± 1.0 | Alcohol 13, HCV 53, alcohol+HCV 23, HBV 16, NASH 8, other 3 | NR | 54/38/21/3 | TACE/TARE 56, RFA 21, combined treatment 15, end-stage life support 24 | CT based SMI at L3 | Martin | Male: 53 cm^2^/m^2^, female: 41 cm^2^/m^2^ | 35(30.17) | OS | 2.53(1.35-4.73) | Sodium, ascites, TNM stage |
| Nishikawa et al. 2017 [37] | Retrospective | Japan | 2009-2015 | 232(181/51) | 72 (40-91) | HBV 33, HCV 144, HBV+HCV 4, viral negative 49, unknown 2 | NR | 1/18/79/134 | unresectable HCC undergoing sorafenib | CT based SMI at L3 | Original | Male: 36.2 cm^2^/m^2^, female: 29.6 cm^2^/m^2^ | 151(65.09) | OS | 2.740(1.938-3.922) | Extrahepatic metastases, tumor burden ≥50%, ascites |
|  |  |  |  |  |  |  |  |  |  |  |  |  |  | PFS | 1.203(0.890-1.634) | NR |
| Salman et al. 2021 [53] | Prospective | Egypt | 2015-2018 | 97(72/25) | 53.4 ± 6.0 | HCV | NR | NR | HCC undergoing RFA | CT based SMI at L3 | Martin | Male: 53 cm^2^/m^2^, female: 43 cm^2^/m^2^ | 42(43.30) | OS | 7.59(3.07-18.77) | MELD socre >9 |
| Shiba et al. 2018 [38] | Retrospective | Japan | 2010-2016 | 68(41/27) | NR | NR | NR | 57/7/4/0 | HCC treated with carbon ion radiotherapy | CT based SMI at L3 | Vledder | Male: 43.75 cm^2^/m^2^, female: 41.10 cm^2^/m^2^ | 22(32.35) | OS | 1.35(0.53-3.22) | NR |
|  |  |  |  |  |  |  |  |  |  |  |  |  |  | PFS | 0.77(0.35-1.53) | NR |
| Shirai et al. 2018 [39] | Retrospective | Japan | 2005-2015 | 402(325/77) | 67.4 ± 9.5 | HBV 264, HCV 138 | NR | 55/179/116/47 | HCC undergoing hepatectomy | CT based PMI at L3 | Original | Male: 6.36 cm^2^/m^2^, female: 3.92 cm^2^/m^2^ | 134(33.33) | OS | 1.176(0.843-1.625) | NR |
| Takada et al. 2020 [40] | Retrospective | Japan | 2011-2018 | 153(114/39) | 73.1 ± 9.2 | HBV or HCV 100, alcohol 19, NASH 33, other 1 | NR | 26/62/32/33 | HCC | CT based PMI at L3 | JSH | Male: 6.0 cm^2^/m^2^, female: 3.4 cm^2^/m^2^ | 43(28.10) | OS | 1.9(1.1-3.4) | JIS score, AFP, PIVKA-II |
| Takagi et al. 2016 [41] | Retrospective | Japan | 2007-2013 | 254(207/47) | 65.7 ± 10.5 | HBV and/or HCV 171, other 83 | NR | 28/110/83/33 | HCC undergoing hepatectomy | CT based SMI at L3 | Martin | Male: 46.4 cm^2^/m^2^, female: 37.6 cm^2^/m^2^ | 118(46.46) | OS | 2.28(1.34-4.01) | ASA grade, tumor number, MVI |
| Tan et al. 2021 [50] | Retrospective | China | 2009-2015 | 70(70/0) | 41.6 ± 8.9 | HBV 60, alcohol 1, other 9 | NR | NR | HCC recipients after living donor liver transplantation | CT based PMI at L3 | Original | Male: 6.25 cm^2^/m^2^, female: 2.88 cm^2^/m^2^ | 38(54.29) | OS | 2.94(1.04-8.30) | Pre-transplant hospital stay |
| Uojima et al. 2020 [14] | Retrospective | Japan | 2018-2019 | 100(75/25) | 71.5 ± 9.2 | HBV 19, HCV 34, alcohol 24, NASH 16, other 7 | 0/0/49/51/0 | NR | HCC treated with lenvatinib | CT based SMI at L3 | JSH | Male: 46.4 cm^2^/m^2^, female: 37.6 cm^2^/m^2^ | 59(59.00) | OS | 2.246(1.091-4.623) | AFP, ALBI score |
| Valero 3rd et al. 2015 [59] | Retrospective | USA | 2000-2013 | 96(59/37) | 61.9 ± 12.3 | HBV 10, HCV 28, viral negative 56, HBV+HCV 2 | NR | 42/34/9/11 | HCC undergoing hepatectomy or liver transplantation | CT based TPV at L3 | Original | Male: 34.14 cm3/m, female: 22.93 cm3/m | 47(48.96) | OS | 1.34(0.61-2.76) | NR |
| Voron et al. 2015 [60] | Retrospective | France | 2006-2012 | 109(92/17) | 61.66 ± 13.30 | Alcohol 12, HBV 22, HCV 27, NASH 11, multifactorial 8, unknown 29 | NR | NR | HCC undergoing hepatectomy | CT based SMI at L3 | Martin | Male: 52.4 cm^2^/m^2^, female: 38.9 cm^2^/m^2^ | 59(54.13) | OS | 3.19(1.28-7.96) | ASA score, involvement of adjacent organ |
|  |  |  |  |  |  |  |  |  |  |  |  |  |  | PFS | 3.03(1.67-5.49) | Age>60, involvement of adjacent organ, satellite nodule |
| Yeh et al. 2020 [51] | Retrospective | China | 2012-2013 | 136(78/58) | 65.34 ± 9.61 | HBV 50, HCV 74, alcohol 12 | 44/92/0/0/0 | NR | HCC undergoing RFA | CT based PMI at L3 | Original | Male: 4.24 cm^2^/m^2^, female: 2.50 cm^2^/m^2^ | 22(16.18) | OS | 2.11(1.092-4.078) | NR |
| Yoshio et al. 2021 [42] | Retrospective | Japan | 2001-2015 | 234(183/51) | 67.4 ± 0.7 | HBV 61, HCV 86, NAFLD 74, other 13 | NR | 81/60/40/53 | HCC undergoing hepatectomy | CT based SMI at L3 | JSH | Male: 42 cm^2^/m^2^, female: 38 cm^2^/m^2^ | 82(35.04) | OS | 1.75(0.62-4.99) | NR |

AFP, alpha-fetoprotein; ALBI, albumin-bilirubin; ASA, American Society of Anesthesiologists; BCLC, Barcelona-Clínic Liver Cancer; BMI, body mass index; CI, confidence interval; CT, computerized tomography; DCP, des-γ-carboxyprothrombin; ECOG PS, Eastern Cooperative Oncology Group performance status; EWGSOP, European Working Group on Sarcopenia in Older People; FFMA, fat-free muscle area; HBV, hepatitis B virus; HCC, hepatocellular carcinoma; HCV, hepatitis C virus; HR, hazard ratio; ICGR15, indocyanine green dye retention test at 15 min; IMAC, intramuscular adipose tissue content; JIS, Japan integrated staging; JSH, Japan Society of Hepatology; L3, third lumbar vertebra; MELD, model for end stage liver disease; MRI, magnetic resonance imaging; MVI, microvascular invasion; NASH, non-alcoholic steatohepatitis; NR, not reported; OS, overall survival; PFS, progression free survival; PIVKA-II, protein induced by vitamin K absence or antagonists-II; PMI, psoas muscle index; SMI, skeletal muscle index; RFA, radiofrequency ablation; SFMI, subcutaneous fat mass index; TACE, transarterial chemoembolization; TAE, transcatheter arterial embolization; TAI, transcatheter arterial infusion; TARE, transarterial radioembolization; TPMT/BH, transverse psoas muscle thickness per body height; TPV, total psoas volume; USA, United States of America; VSR, visceral-to-subcutaneous adipose tissue area.

*Data presented as mean ± SD or median (range).

^#^HR estimated by Cox Proportional-Hazards Regression

**Supplementary Table 3.** Risk of bias of included studies using the Quality in Prognosis Studies tool

| Study | Study participation | Study attrition | Prognostic factor measurement | Outcome measurement | Adjustment for other prognostic factors | Statistical analysis and reporting | Overall rating |
| --- | --- | --- | --- | --- | --- | --- | --- |
| Alsebaey et al. 2021 [52] | moderate | low | low | low | low | low | **LOW** |
| Antonelli et al. 2018 [55] | moderate | moderate | low | low | low | low | **LOW** |
| Cheng et al. 2020 [48] | moderate | low | low | low | moderate | moderate | **MODERATE** |
| Choi et al. 2020 [43] | low | low | low | low | low | low | **LOW** |
| Dong et al. 2022 [49] | moderate | moderate | low | low | low | low | **LOW** |
| Endo et al. 2020 [22] | moderate | low | low | low | low | low | **LOW** |
| Faron et al. 2020 [54] | moderate | low | low | low | low | low | **LOW** |
| Fujita et al. 2019 [23] | moderate | low | low | low | low | moderate | **LOW** |
| Fujiwara et al. 2015 [24] | moderate | low | low | low | low | low | **LOW** |
| Ha et al. 2018 [16] | moderate | low | low | low | low | low | **LOW** |
| Hamaguchi et al. 2015 [25] | moderate | low | low | low | low | low | **LOW** |
| Hamaguchi et al. 2020 [26] | moderate | moderate | low | low | moderate | low | **MODERATE** |
| Harimoto et al. 2013 [27] | moderate | moderate | low | low | moderate | low | **MODERATE** |
| Harimoto et al. 2016 [28] | moderate | low | low | low | low | low | **LOW** |
| Hiraoka et al. 2017 [29] | moderate | low | low | low | low | moderate | **LOW** |
| Hiraoka et al. 2018 [30] | moderate | low | low | low | low | low | **LOW** |
| Hiraoka et al. 2021 [31] | moderate | low | low | low | low | low | **LOW** |
| Imai et al. 2019 [32] | moderate | low | low | low | low | low | **LOW** |
| Iritani et al. 2014 [33] | moderate | moderate | low | low | moderate | low | **MODERATE** |
| Itoh et al. 2014 [34] | moderate | low | low | low | low | low | **LOW** |
| Jang et al. 2021 [44] | moderate | low | low | low | low | low | **LOW** |
| Kim et al. 2021 [45] | moderate | moderate | low | low | moderate | low | **MODERATE** |
| Kobayashi et al. 2018 [35] | moderate | low | low | low | moderate | low | **LOW** |
| Lanza et al. 2020 [56] | moderate | low | low | low | low | low | **LOW** |
| Lee et al. 2019 [46] | moderate | low | low | low | low | low | **LOW** |
| Levolger et al. 2015 [57] | moderate | low | low | low | low | low | **LOW** |
| Lim et al. 2021 [47] | moderate | low | low | low | low | low | **LOW** |
| Loosen et al. 2019 [15] | low | low | low | low | low | low | **LOW** |
| Mardian et al. 2019 [36] | moderate | moderate | low | low | moderate | low | **MODERATE** |
| Meza-Junco et al. 2013 [58] | low | low | low | low | low | low | **LOW** |
| Nishikawa et al. 2017 [37] | moderate | low | low | low | low | low | **LOW** |
| Salman et al. 2021 [53] | low | low | low | low | low | low | **LOW** |
| Shiba et al. 2018 [38] | moderate | low | low | low | moderate | moderate | **MODERATE** |
| Shirai et al. 2018 [39] | moderate | low | low | low | low | low | **LOW** |
| Takada et al. 2020 [40] | moderate | low | low | low | low | low | **LOW** |
| Takagi et al. 2016 [41] | moderate | low | low | low | low | low | **LOW** |
| Tan et al. 2021 [50] | moderate | low | low | low | low | low | **LOW** |
| Uojima et al. 2020 [14] | moderate | low | low | low | low | low | **LOW** |
| Valero 3rd et al. 2015 [59] | moderate | low | low | low | low | low | **LOW** |
| Voron et al. 2015 [60] | moderate | moderate | low | low | low | moderate | **MODERATE** |
| Yeh et al. 2020 [51] | moderate | low | low | low | low | low | **LOW** |
| Yoshio et al. 2021 [42] | moderate | low | low | low | low | low | **LOW** |
